# Polyphenols of *Salix aegyptiaca* modulate the activities of drug metabolizing and antioxidant enzymes, and level of lipid peroxidation

**DOI:** 10.1186/s12906-018-2143-7

**Published:** 2018-03-07

**Authors:** Mohd Nauman, R. K. Kale, Rana P. Singh

**Affiliations:** School of Life Sciences, Jawaharlal Nehru University, New Delhi, 110067 India

**Keywords:** Polyphenols, Redox potential, Antioxidant activity, Anti-inflammatory activity, Phase I and phase II enzymes, Antioxidant enzymes

## Abstract

**Background:**

*Salix aegyptiaca* is known for its medicinal properties mainly due to the presence of salicylate compounds. However, it also contains other beneficial phytochemicals such as gallic acid, quercetin, rutin and vanillin. The aim of the study was to examine the redox potential, antioxidant and anti-inflammatory activity of these phytochemicals along with acetylsalicylic acid.

**Methods:**

The redox potential and antioxidant activity of gallic acid, quercetin, rutin, vanillin and acetylsalicylic acid were determined by oxidation-reduction potential electrode method and 1,1-diphenyl-2-picrylhydrazyl (DPPH) assay, respectively. In *ex vivo* studies, antioxidant activity of these phytochemicals was determined by lipid peroxidation and carbonyl content assay in the liver of mice. Anti-inflammatory activity was determined by protein denaturation method. Six-week old C57BL/6 mice treated with gallic acid (100 mg/kg body weight) and acetylsalicylic acid (25 and 50 mg/kg body weight) to investigate their *in vivo* modulatory effects on the specific activities of drug metabolizing phase I and phase II enzymes, antioxidant enzymes and level of lipid peroxidation in liver.

**Results:**

The order of ability to donate electron and antioxidant activity was found to be: gallic acid > quercetin > rutin > vanillin > acetylsalicylic acid. In *ex vivo* studies, the similar pattern and magnitude of inhibitory effects of these phytochemicals against peroxidative damage in microsomes and protein carbonyl in cytosolic fraction were observed. In *in vivo* studies, gallic acid and acetylsalicylic acid alone or in combination, enhanced the specific activities of drug metabolizing phase I and phase II enzymes as well as antioxidant enzymes and also inhibited lipid peroxidation in liver.

**Conclusions:**

These findings show a close link between the electron donation and antioxidation potential of these phytochemicals, and in turn their biological activity. Gallic acid, quercetin, rutin and vanillin were found to be better electron donors and antioxidants and therefore, might be mainly responsible for the antioxidant properties of *S. aegyptiaca*, while acetylsalicylic acid provided its maximum anti-inflammatory activity.

## Background

*S. aegyptiaca*, a deciduous plant, belongs to *salicaceae* family. It is popularly known as Musk Willow and mainly cultivated in Middle-East region of the world [1]. Apart from it’s use in confectionary, flavourful syrup and fragrance additive, it has been part of traditional medicine from ancient time. The extract from different parts of this plant such as bark and leaves have shown to exert beneficial effects, as laxative, cardioprotective, nervonic, sedative, hypnotic, somnolent, aphrodisiac, orexoiogenic, carnative, gastroprotector, anthelmintic and vermifuge [2].

The *salicaceae* family, contains salicylate compounds including salicylic acid which subsequently lead to the discovery of acetylsalicylic acid known as aspirin, which is used throughout the world as analgesic, antipyretic, anti-inflammatory drug [3]. Expectedly, the rich presence of salicylates generated interest to find out the beneficial effects of *S. aegyptiaca* and other species of *salicaceae* family. However, pharmacological studies indicated that its beneficial effects could not be adequately ascribed to salicylates [4], and suggested the possible contribution from other antioxidant phytochemicals [5]. Subsequently, the presence of other polyphenols such as gallic acid, caffeic acid, vanillin, p-coumaric acid, myricetin, catechin, epigallocatechin gallate, rutin and quercetin etc., were confirmed by the analytical studies which would be contributing to the beneficial effects of *S. aegyptiaca* [6].

Oxidative stress could lead to the initiation and development of several health complications such as diabetes, Alzheimer’s disease, atherosclerosis, cardiovascular problems and various kinds of cancers [7, 8]. Considering the wide range of medicinal applications of *S. aegyptiaca*, it would be interesting and essential to understand the bio-activeness of its flavonoid and phenolic phytochemicals other than salicylates, using various biological end points. Such phytochemicals with antioxidant activity are likely to maintain redox homeostasis, disturbed on generation of products of cellular mechanism or the consequences of exposure to detrimental chemical agents; by scavenging the free radicals, influencing the antioxidant non-enzymatic and enzymatic defense systems as well as drug metabolising enzyme systems. In turn, these phytochemicals are expected to modulate the diverse biological activities such as inflammation, necrosis and carcinogenesis leading to cytoprotection. With this backdrop, an attempt was made to examine the interdependency of redox-potential, anti-oxidant activity and anti-inflammatory activity of gallic acid, quercetin, rutin and vanillin as well as acetylsalicylic acid. Further, to find the relevance of above results in the biological systems, the influence of gallic acid and acetylsalicylic acid has been studied on the drug metabolising phase I and phase II enzymes as well as on endogenous antioxidant enzymes and peroxidative damage in the liver of C57BL/6 mice.

## Methods

### Chemicals

Gallic acid, vanillin, acetylsalicylic acid, quercetin, rutin, 3,5-Di-*tert*-4-butylhydroxytoluene (BHT), ascorbic acid, sodium diclofenac, 1,1-Diphenyl-2-picrylhydrazyl (DPPH), guanidine hydrochloride,1-chloro-2,4-dinitrobenzene (CDNB), 5,50-dithiobis-2-nitrobenzoic acid (DTNB), reduced glutathione (GSH), oxidized GSH (GSSG), pyrogallol, 2,6-dichlorophenol-indophenol (DCPIP), potassium ferricyanide, triton X-100, ethylenediaminetetraacetic acid (EDTA), sodium pyruvate, thiobarbituric acid (TBA), reduced nicotinamide adenine dinucleotide (NADH) and reduced nicotinamide adenine dinucleotide phosphate (NADPH) were obtained from Sigma Chemical Co. (St. Louis, MO, USA). The rest of the chemicals used were procured from local firms (India) and were of highest purity grade.

### Investigation of oxidation-reduction potential

The oxidation-reduction potential (ORP) of phytochemicals was determined according to the modified method of M. Liu [9] using ORP electrode (Hanna instruments, USA). Phosphate buffer saline (PBS) was used as reference solution. The phytochemicals namely gallic acid, vanillin, acetylsalicylic acid, quercetin and rutin dissolved in dimethyl sulphoxide (DMSO) were mixed in PBS with varying concentrations (5–50 μg/ml). The reduction potential was measured in milivolt (mV).

### Determination of antioxidant activity

The free radical scavenging activity of phytochemicals was measured by DPPH method as described by G. C. Yen [10] with slight modification. Briefly, 1.3 ml of methanolic solution of DPPH (100 μM) was added to varying concentrations of phytochemicals (2–10 μg/ml). The reaction was incubated at room temperature for 30 min in dark. The absorbance of the residual DPPH solution was determined at 517 nm in a UV-1800 Spectrophotometer (Shimadzu Corp). Butylhydroxytoluene (BHT) was used as positive control. The percent DPPH scavenging effect was calculated using following formula,

% DPPH scavenging effect = [(Abs_control_ – Abs_sample_) / Abs_control_] × 100

Where, Abs_control_ is the absorbance of DPPH radical + methanol and Abs_sample_ is absorbance of DPPH radical + phytochemical/standard.

### Determination of anti-inflammatory activity

The anti-inflammatory activity was studied by the method of H. M. Arif Ullah [11]. The final reaction mixture contained 0.2 ml of egg albumin, 2.8 ml of phosphate buffer saline (PBS) and 2 ml of varying concentrations of the phytochemicals (62.5–1000 μg/ml). Sodium diclofenac was used as a standard. The tubes containing the mixture were incubated at 37 °C for 15 min and heated at 70 °C for 5 min. On cooling, the absorbance was measured at 660 nm. The percent inhibition of protein denaturation was calculated by using the following formula,

% Inhibition of protein denaturation = [(Abs_control_ – Abs_sample_) / Abs_control_] × 100

Where, Abs_control_ is the absorbance of control and Abs_sample_ is absorbance of phytochemical/standard.

### Animals

In the present study, six weeks old, male C57BL/6 mice were used for *ex vivo* and *in vivo* experiments. The animals were provided by Central Laboratory Animal Resources, Jawaharlal Nehru University, New Delhi, India. The animals were kept in polypropylene cages in a room maintained with controlled temperature (22 °C ± 1), 60–70% humidity and a 12 h light/12 h dark cycle and provided with standard food pellets and drinking water ad libitum, in Central Laboratory Animal Resources, Jawaharlal Nehru University, New Delhi. The animals were divided randomly into the groups and kept under observation throughout the duration of experimentation, in terms of body weight, food and water consumption, and for any sign of health toxicity. All the mice were euthanized by CO_2_ asphyxiation in CO_2_ chamber. The experiments were approved by the Committee for the Purpose of Control and Supervision of Experiments on Animals (CPCSEA), Government of India and Jawaharlal Nehru University Institutional Animal Ethics Committee (IEAC). The experiments were carried out as per their guidelines.

### Ex vivo studies

The free radical scavenging activity of the phytochemicals was determined in an *ex vivo* system as described by B. Uddin [12]. The animals were euthanized, livers were excised and perfused with 0.9% saline. The livers were blot dried and 20% *w*/*v* liver tissue homogenate (LH) was prepared with phosphate buffer (25 mM, pH 7.4) using electric homogenizer. Fenton reagent (0.5 mM FeSO_4_: 0.5 mM H_2_O_2_; 1:1) was used to generate free radicals. Control group contained 4 ml liver homogenate (LH) + 8 ml double distilled water (DDW), positive control group contained 4 ml LH + 4 ml Fenton reagent + 4 ml DDW, while the rest group were mixed with standard (GSH) and phytochemicals namely gallic acid, quercetin, rutin, vanillin and acetylsalicylic acid, giving the final reaction mixture as 4 ml LH + 4 ml Fenton reagent + 4 ml of drug with varying concentrations (62.5–1000 μg/ml). The test tubes of reaction mixture were incubated for four hours at 37 °C using water bath. The tubes were centrifuged at 10,000 rpm for 20 min. The resultant supernatant was again centrifuged at 105,000×g for 60 min in a Beckman ultracentrifuge. The cytosolic fraction (supernatant) was used for the estimation of protein carbonyl content whereas the pellet representing microsomes was used to determine the peroxidative damage.

### Estimation of protein carbonyl content

Protein carbonyl was estimated according to method of J. M. Fagan [13]. An aliquot of 500 μl cytosolic fraction was treated with an equal volume of 10 mM of 2,4-dinitrophenylhydrazine (DNPH) dissolved 2 M HCl and incubated at room temperature. After 1 h, the tubes were vortexed and 500 μl of 30% TCA was added to each tube and incubated on ice for 15 min. The tubes were centrifuged at 11,000 g for 15 min. The supernatant was discarded and the resulting pellet was treated with 1 ml of ethanol:ethylacetate (1:1) solution. The pellet was vortexed, kept for 15 min and centrifuged again at 11,000 g for 15 min. The supernatant was discarded and pellet again washed with ethanol-ethyl acetate solution two more times following the same steps to remove out the excessive DNPH. The pellet was dissolved in 6 M guanidium hydrochloride prepared in 20 mM potassium dihydrogen phosphate buffer (pH 2.3). The sample with no DNPH and dissolved in guanidium hydrochloride used as blank corresponding to their sample with DNPH. The absorbance was recorded at 380 nm. The protein carbonyl formation was expressed as moles of dinitrophenyl hydrazine (DNPH) incorporated/100 mg protein using a molar extinction coefficient of 21 mM^− 1^ cm^− 1^.

### Estimation of peroxidative damage

The microsomes were resuspended into homogenizing buffer and used to study the peroxidative damage by thiobarbituric acid reactive substances (TBARS) method as described by Varshney and Kale [14]. The peroxidative damage was expressed in terms of malondialdehyde (MDA) formed per mg protein. In brief, 0.5 ml of microsomal fraction was mixed with 1.5 ml of 0.15 M Tris-KCl buffer (pH 7.4), 0.5 ml of 30% TCA and 0.5 ml of 52 mM thiobarbituric acid (TBA). The tubes were kept in a water bath for 45 min at 80 °C, cooled in ice and centrifuged for 10 min at 3000 rpm. The absorbance of the clear supernatant was measured at 531.8 nm.

### In vivo studies

Modulatory effects of gallic acid (100 mg/kg body weight) and acetylsalicylic acid (25 & 50 mg/kg body weight), alone as well as in combination, were determined on the specific activities of the enzymes involved in drug metabolizing and antioxidant function as well as the level of peroxidative damage in the liver of mice. 25 and 50 mg/kg body weight of acetylsalicylic acid in mice are within the range of its therapeutic doses which are given as analgesic and cardioprotectant purposes to human [15]. In case of gallic acid, a dose of 100 mg/kg bodyweight was reported to be non-toxic [16], effective and widely used [17].

### Animal grouping and treatments

A suspension of gallic acid and acetylsalicylic acid was made in 0.5% carboxymethyl cellulose (CMC) and fed orally to mice using a gavage at alternate days for two weeks [18–20]. After completion of dosing the mice were euthanized, livers were excised and liver tissue homogenate was prepared. The experimental groups were designed as shown in Table 1.

**Table 1:** Experimental design to determine the modulation of the specific activities of drug metabolizing enzymes and endogenous antioxidant enzymes, as well as the level of peroxidative damage

*Group*	*Number of animals*	*Treatment (mg/kg body weight)*
I	6	Control, only 0.5% (w/v) CMC as vehicle
II	6	25 acetylsalicylic acid
III	6	50 acetylsalicylic acid
IV	6	100 gallic acid
V	6	25 acetylsalicylic acid + 100 gallic acid
VI	6	50 acetylsalicylic acid + 100 gallic acid

### Preparation of homogenate, cytosol and microsomes

After the completion of two weeks treatment the animals were sacrificed and their liver were perfused immediately with ice-cold NaCl (0.9%) and removed carefully, thereafter rinsed in chilled 0.15 M of Tris-KCl buffer (pH 7.4). The livers were then blot dried, weighed quickly and homogenized in ice cold 0.15 M Tris-KCl buffer (pH 7.4) to yield 10% (*w*/*v*) homogenate. The microsomes and cytosolic fraction was prepared from the homogenate as described earlier in *ex vivo* section. The specific activities of glutathione-S-transferase (GST), DT-diaphorase (DTD), and antioxidant enzymes were determined in cytosolic fraction. The specific activities of cytochrome P450 reductase, cytochrome b5 reductase and extent of peroxidative damage were measured in microsomes.

### Determination of NADPH-cytochrome P450 reductase and NADH-cytochrome b5 reductase activities

The specific activity of NADPH-cytochrome P450 reductase was determined according to Omura and Takesue [21] with some modifications, measuring the rate of oxidation of NADPH at 340 nm. The reaction mixture was consisted of 0.3 M potassium phosphate buffer (pH 7.5), 0.1 mM NADPH, 0.2 mM potassium ferricyanide and microsomal fraction. The enzyme activity was calculated using extinction coefficient 6.22 mM^− 1^ cm^− 1^. One unit of enzyme activity is defined as that causing the oxidation of 1 mol of NADPH per minute. The specific activity of NADH-cytochrome b5 reductase was measured following the method of Mihara and Sato [22] with some modifications, measuring the rate of reduction of potassium ferricyanide at 420 nm by NADH. The reaction mixture consisted of 0.1 M potassium phosphate buffer (pH 7.5), 0.1 mM NADH, 1 mM potassium ferricyanide and microsomal fraction. The enzyme activity was calculated using the extinction coefficient of 1.02 mM^− 1^ cm^− 1^. One unit of enzyme activity is defined as that causing the reduction of 1 mol of ferricyanide per minute.

### Determination of glutathione S-transferase and DT-diaphorase activities

The specific activity of cytosolic GST was determined according to the method of Habig et al. [23]. The reaction mixture was consisted of 0.1 M phosphate buffer (pH 6.5), 1 mM CDNB in 95% ethanol and 1 mM GSH and was incubated at 37 °C for 5 min and absorbance was recorded at 340 nm. The specific activity was expressed as micromoles of GSH-CDNB conjugate formed/min/mg protein using the extinction coefficient 9.6 mM^− 1^ cm^− 1^. The specific activity of DTD was determined by carried out the method of Ernster et al. [24]. NADH was used as the electron donor and DCPIP as the electron acceptor at 600 nm. The reaction mixture was containing 50 mM Tris–HCl buffer (pH 7.5), 0.5 mM NADH, 40 μM DCPIP and 0.08% Triton X-100. The activity was calculated using extinction coefficient 21 mM^− 1^ cm^− 1^. One unit of enzyme activity is defined as amount of enzyme required to reduce one micromole of DCPIP per minute.

### Determination of superoxide dismutase and catalase activities

The specific activity of SOD was determined following the method of Marklund and Marklund [25] that involves the inhibition of autooxidation of pyrogallol at pH 8.0. A single unit of enzyme was defined as the quantity of superoxide dismutase required to produce 50% inhibition of autooxidation. The cytosolic fraction was treated with Triton X-100 (1%) and kept at 4 °C for 30 min then added to the assay mixture that contained 0.05 M sodium phosphate buffer (pH 8.0), 0.1 mM EDTA and 0.27 mM pyrogallol. The absorbance was recorded at 420 nm for 5 min. The specific activity of catalase was determined according to the method of Aebi [26], by analyzing the disappearance of H_2_O_2_. The cytosolic fraction was treated with Triton X-100 (1%) and ethanol (10 μl/ml) and then incubated in ice for 30 min. This reaction mixture was added to 0.05 M sodium phosphate buffer (pH 7.0) and 10 mM H_2_O_2_. The decrease in absorbance was measured at 240 nm. The activity was calculated using the extinction coefficient as 40 μmol^− 1^ cm^− 1^. The specific activity of catalase is expressed as moles of H_2_O_2_ reduced/min/mg protein.

### Determination of glutathione reductase and glutathione peroxidase activities

The specific activity of GR was determined according to the method published earlier [27]. The reaction mixture contained cytosolic fraction, 0.2 M sodium phosphate buffer (pH 7.0), 2 mM EDTA, 1 mM GSSG and 0.2 mM NADPH. The enzyme activity was measured by analyzing oxidation of NADPH following decrease in absorbance at 340 nm. One unit of enzyme activity was defined as nmoles of NADPH consumed/min/mg protein, based on an extinction coefficient of 6.22 mM^− 1^ cm^− 1^. The specific activity of GPx was determined by the method of Paglia and Valentine [28]. The reaction mixture consisted of cytosolic fraction, 50 mM sodium phosphate buffer (pH 7.0) containing EDTA, 0.24 U/ml yeast glutathione reductase, 0.3 mM reduced glutathione, 0.2 mM NADPH, 1.5 mM H_2_O_2_ and cytosolic. The reaction was initiated by the addition of NADPH and decrease in the absorbance was monitored at 340 nm for 5 min. One unit of enzyme activity has been defined as nmoles of NADPH consumed/min/mg protein based on an extinction coefficient of 6.22 mM^− 1^ cm^− 1^.

### Estimation of peroxidative damage

Lipid peroxidation was estimated in microsomes, following the method of Varshney and Kale (1990) as described in the *ex vivo* section.

### Protein estimation

Protein content of the samples was determined using Bradford’s reagent and BSA is used as standard at 595 nm.

### Statistical analysis

The values are presented as mean ± SEM. The mean and significance of the differences between the data pairs was calculated by ANOVA followed by Tukey’s test. A value of *p* < 0.05 was considered significant.

## Results

### Oxidation-reduction potential

**Fig. 1:**
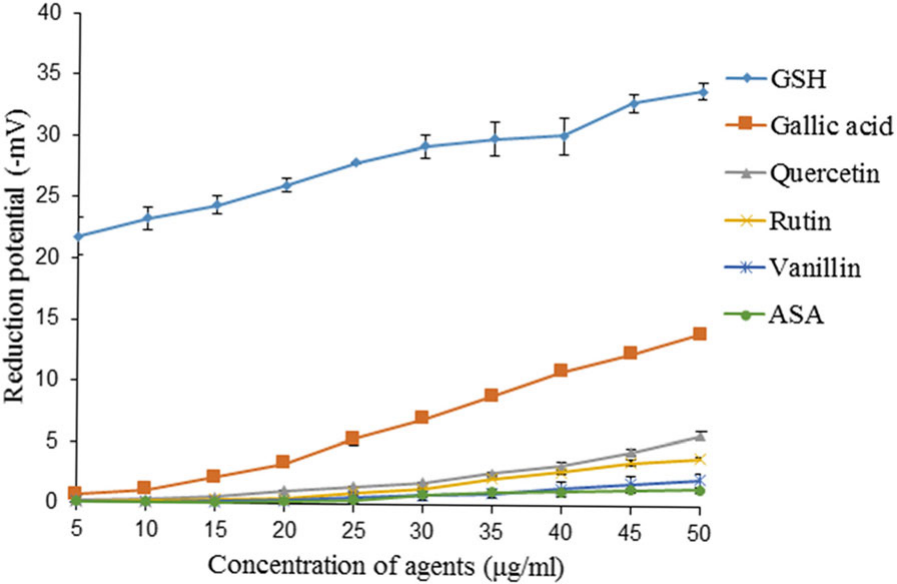
Reduction potential; reduced glutathione, gallic acid, vanillin, acetylsalicylic acid, quercetin and rutin were added at 5–50 μg/ml of phosphate buffer saline (pH 7.4). The reduction potential was measured in milivolt (mV) using ORP electrode. Reduced glutathione was used as a positive control. Values are represented as mean ± SEM of three samples

The oxidation-reduction potential of gallic acid, acetylsalicylic acid, rutin, quercetin and vanillin was tested. All the agents exhibited reduction potential which increased with increase in the concentration from 5 to 50 μg/ml. Gallic acid exhibited the highest reduction potential followed by quercetin, rutin, vanillin and acetylsalicylic acid (Fig. 1).

### DPPH radical scavenging activity

The antioxidant activity of different concentrations (2–10 μg/ml) of gallic acid, acetylsalicylic acid, rutin, quercetin and vanillin was examined on the scavenging of DPPH radicals. All these phytochemicals exhibited the inhibition of DPPH radicals, a measure of antioxidant activity, in concentration dependent manner. When compared, the maximum DPPH radical scavenging activity was shown by gallic acid (IC_50_: 1.88 ± 0.12 μg/ml) followed by quercetin (IC_50_: 2.10 ± 0.06 μg/ml), rutin (IC_50_: 3.10 ± 0.21 μg/ml), vanillin (IC_50_: 68.53 ± 0.74 μg/ml) and acetylsalicylic acid (IC_50_: 132.50 ± 0.81 μg/ml). These results for DPPH radical scavenging activity are shown below:

**Table:** 

*Agents*	*IC*_*50*_ *value (μg/ml)*
BHT	7.69 ± 0.04
Gallic acid	1.88 ± 0.12
Quercetin	2.10 ± 0.06
Rutin	3.10 ± 0.21
Vanillin	68.53 ± 0.74
Acetylsalicylic acid	132.50 ± 0.81

### Anti-inflammatory activity

**Fig. 2:**
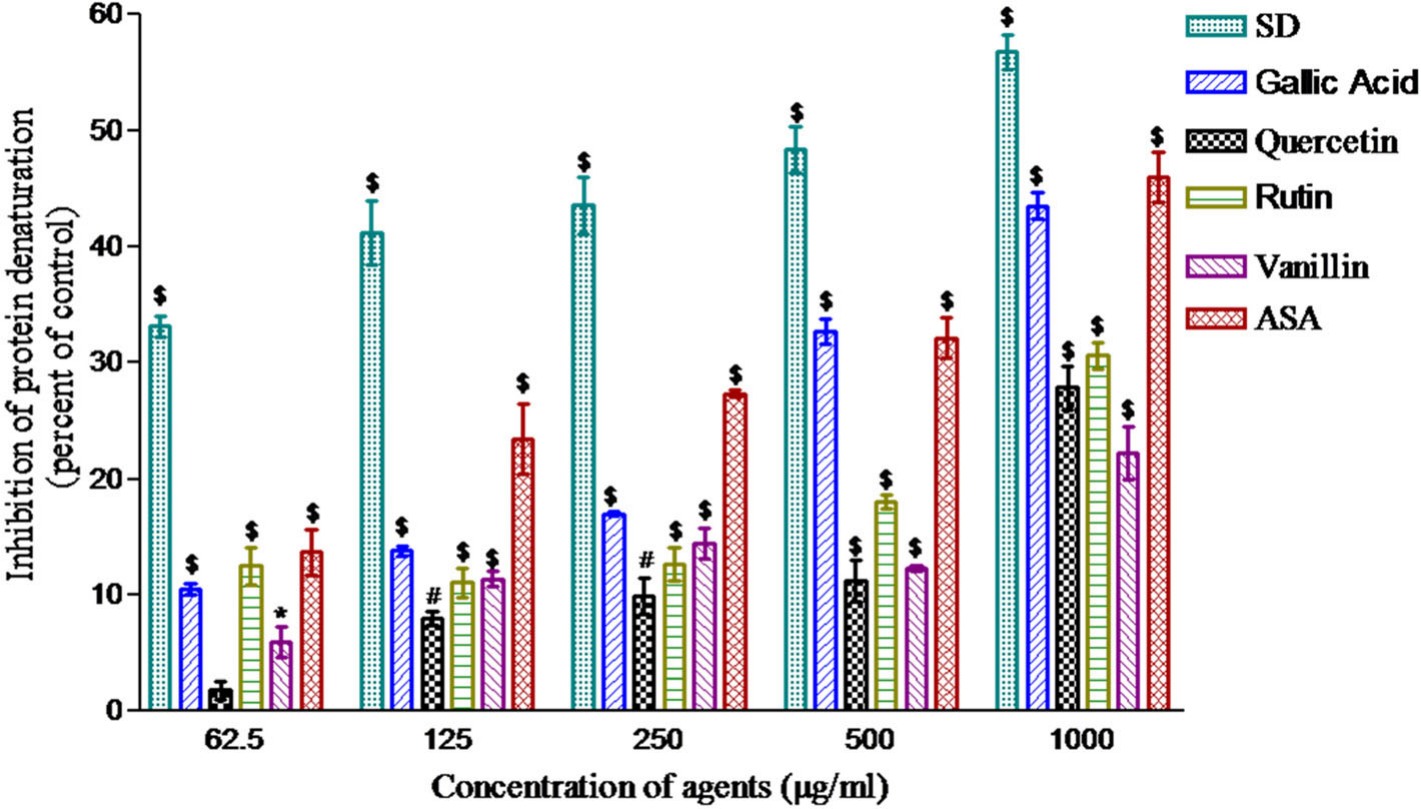
Anti-inflammatory activity; inhibition in the heat induced denaturation of egg albumin protein. Sodium diclofenac, gallic acid, vanillin, acetylsalicylic acid, quercetin and rutin were added at 62.5–1000 μg/ml. The tubes containing reaction mixture were incubated at 37 °C for 15 min and heated at 70 °C for 5 min. The tubes were allowed for cooling and absorbance was measured at 660 nm. Sodium diclofenac was used as a positive control. Values are represented as mean ± SEM of three samples. ^*^(*p* < 0.05), ^#^(*p* < 0.01) and ^§^(*p* < 0.001) represent significant changes relative to control

The anti-inflammatory activity of the phytochemicals (62.5–1000 μg/ml) was determined as inhibition of protein denaturation. Their concentration-dependent inhibitory effect is shown in Fig. 2. The relative inhibitory effect of these phytochemicals was found in the following order: acetylsalicylic acid > gallic acid > rutin > quercetin > vanillin.

### Protein carbonyl estimation

**Fig. 3:**
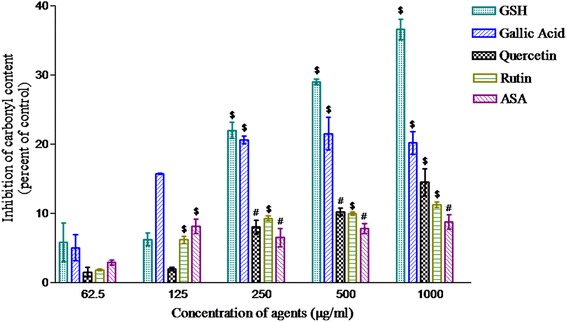
Protein carbonyl content; inhibition in formation of protein carbonyls (nmole carbonyl/mg of protein) against Fenton reagent. Reduced glutathione, gallic acid, quercetin, rutin, vanillin and acetylsalicylic acid were added at 62.5–1000 μg/ml. The test tubes were incubated for four hours at 37 °C followed by two consecutive centrifugations. The supernatant was used for protein carbonyl assay. Reduced glutathione was used as a positive control. Values are represented as mean ± SEM of three samples. ^#^(*p* < 0.01) and ^§^(*p* < 0.001) represent significant changes relative to control

In the present study, gallic acid, acetylsalicylic acid, rutin and quercetin (62.5–1000 μg/ml) showed concentration-dependent protection against protein carbonyl damage caused by the Fenton reagent except vanillin which was excluded from further studies. The percent protection as compared to control was found to be highest for gallic acid followed by quercetin, rutin and acetylsalicylic acid (Fig. 3).

### Peroxidative damage

**Fig. 4:**
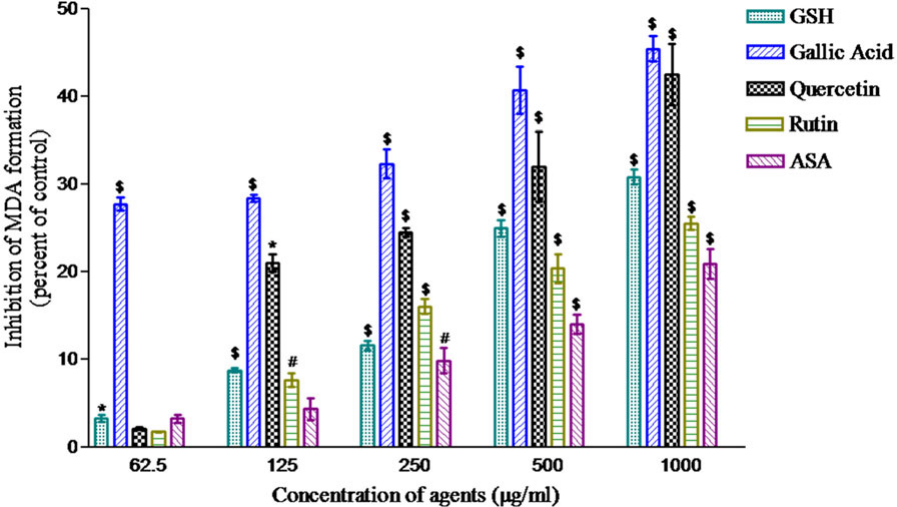
Peroxidative damage; inhibition in the formation of MDA (nmole MDA/mg of protein) against Fenton reagent. Reduced glutathione, gallic acid, quercetin, rutin, vanillin and acetylsalicylic acid were added at 62.5–1000 μg/ml. The test tubes were incubated for four hours at 37 °C followed by two consecutive centrifugations. The resulting microsome was used for peroxidative damage estimation. Reduced glutathione was used as a positive control. Values are represented as mean ± SEM of three samples. ^*^(*p* < 0.05), ^#^(*p* < 0.01) and ^§^(*p* < 0.001) represent significant changes relative to control

Peroxidative damage and its inhibition by phytochemicals (62.5–1000 μg/ml) was studied in the microsomes. Peroxidation was initiated by the Fenton reagent and determined in terms of TBARS formation. Except vanillin, all the phytochemicals showed inhibitory effect against peroxidative damage, in a dose dependent manner. When compared, the order of inhibition was seen to be: gallic acid > quercetin > rutin > acetylsalicylic acid (Fig. 4).

### Cytochrome P450 reductase and cytochrome b5 reductase

A significant increase in the specific activity of cytochrome P450 reductase by 1.26 fold (*p* < 0.01) and 1.45 fold (*p* < 0.001) were observed in the animals of group III, treated with 50 mg/kg body weight of acetylsalicylic acid and group IV treated with 100 mg/kg body weight of gallic acid, respectively as compared to control group. While the animals belonging to group V and VI treated with 100 mg/kg body weight of gallic acid + 25 mg/kg body weight of acetylsalicylic acid, and 100 mg/kg body weight of gallic acid + 50 mg/kg body weight of acetylsalicylic acid showed further enhancement in the specific activity of this enzyme by 1.58 fold (*p* < 0.001) and 1.66 fold (*p* < 0.001) respectively as compared to control group. Group V showed enhancement in the specific activity by 1.36 fold (*p* < 0.01) as compared to 25 mg/kg body weight of acetylsalicylic acid treated group. Group VI showed enhancement in the specific activity by 1.32 fold (*p* < 0.001) and 1.14 fold (0.05) as compared to 50 mg/kg body weight of acetylsalicylic acid and 100 mg/kg body weight of gallic acid, respectively. The specific activity of cytochrome b5 reductase exhibited significant increase in the animals of group III, treated with 50 mg/kg body weight dose of acetylsalicylic acid and the animals from group IV treated with gallic acid (100 mg/kg body weight) by 1.47 fold (*p* < 0.01) and 1.48 fold (*p* < 0.01), respectively as compared to control. The animals of groups V and VI treated 100 mg/kg body weight of gallic acid + 25 mg/kg body weight of acetylsalicylic acid, and 100 mg/kg body weight of gallic acid + 50 mg/kg body weight of acetylsalicylic acid exhibited an increase in the specific activity of this enzyme by 1.72 fold (*p* < 0.001) and 1.72 fold (*p* < 0.001), respectively as compared to control. Group V showed enhancement in the specific activity by 1.39 fold (*p* < 0.01) as compared to 25 mg/kg body weight of acetylsalicylic acid. The combined inductive effects of gallic acid and acetylsalicylic acid were more than their individual effect on the specific activity of both of the enzymes. The results are depicted in Table 2.

**Table 2:** Inductive effects of gallic acid, acetylsalicylic acid and their combinations on the specific activities of hepatic phases I and II drug metabolizing enzymes in mice

Groups→ Parameters ↓	Control	25 mg/kg acetylsalicylic acid	50 mg/kg acetylsalicylic acid	100 mg/kg gallic acid	100 mg/kg gallic acid + 25 mg/kg acetylsalicylic acid	100 mg/kg gallic acid + 50 mg/kg acetylsalicylic acid
Cyt P450 R (μmole of NADPH oxidized/min/mg protein)	0.183 ± 0.002 (100)	0.214 ± 0.014 (116.67)	0.231 ± 0.004^#^ (126.26)	0.267 ± 0.009^§^ (145.61)	0.291 ± 0.004^§€^ (158.58)	0.306 ± 0.003^§ȣɛ^ (166.86)
Cyt b5 R (μmole of NADH oxidized/min/mg protein)	2.030 ± 0.205 (100)	2.521 ± 0.197 (124.13)	3.001 ± 0.012^#^ (147.81)	3.015 ± 0.184^#^ (148.50)	3.510 ± 0.097^§€^ (172.87)	3.508 ± 0.006^§^ (172.75)
GST (μmole of CDNB-GSH conjugate formed/min/mg protein)	1.246 ± 0.02 (100)	1.742 ± 0.021 (139.80)	2.399 ± 0.176^#^ (192.52)	2.641 ± 0.257^§^ (211.96)	2.809 ± 0.167^§€^ (225.43)	3.215 ± 0.150^§¥^ (257.82)
DTD (μmole of DCPIP reduced/min/mg protein)	0.035 ± 0.002 (100)	0.043 ± 0.002 (121.36)	0.063 ± 0.003^§^ (179.48)	0.083 ± 0.002^§^ (236.46)	0.084 ± 0.001^§£^ (241.88)	0.092 ± 0.002^§ȣɛ^ (264.38)

Gallic acid and acetylsalicylic acid were given alone and in combinations through oral gavage for two weeks at desired doses (milligrams of per kilogram of body weight) as mentioned in the table. Abbreviations: *Cyt P450 R* cytochrome P450 reductase, *Cyt b5 R* cytochrome b5 reductase, *GST* glutathione S-transferase, *DTD* DT-diaphorase. Values are expressed as mean ± SEM of 5–6 animals. Values in parentheses are presented as percent of control. ^#^(*p* < 0.01) and ^§^(*p* < 0.001) represent significant changes relative to control. ^€^(*p* < 0.01) and ^£^(*p* < 0.001) represent significant changes relative to 25 mg/kg body weight of acetylsalicylic acid. ^¥^(*p* < 0.05) and ^ȣ^(*p* < 0.001) represent significant changes relative to 50 mg/kg body weight of acetylsalicylic acid. ^ɛ^(*p* < 0.05) represents significant changes relative to 100 mg/kg body weight of gallic acid

### Glutathione S-transferase and DT-diaphorase

The mice treated with 50 mg/kg body weight of acetylsalicylic acid (group III) and 100 mg/kg body weight of gallic acid (group IV) showed significant increase in the specific activity of glutathione S-transferase (GST) by 1.92 fold (*p* < 0.01) and 2.11 fold (*p* < 0.001) as compared to control group. Expectedly, the group V and VI treated with 100 mg/kg body weight of gallic acid + 25 mg/kg body weight of acetylsalicylic acid, and 100 mg/kg body weight of gallic acid + 50 mg/kg body weight of acetylsalicylic acid showed relatively higher elevation in the specific activity of this enzyme by 2.25 fold (*p* < 0.001) and 2.57 fold (*p* < 0.001) compared to control group. Group V showed enhancement in the specific activity by 1.16 fold (*p* < 0.01) as compared to 25 mg/kg body weight of acetylsalicylic acid. Group VI exhibited enhancement in the specific activity by 1.21 fold (*p* < 0.05) as compared to 100 mg/kg body weight of gallic acid. In the case of DT-diaphorase (DTD), its specific activities were found to be significantly elevated by 1.79 fold (*p* < 0.001) and 2.36 fold (*p* < 0.001), in the group III treated with 50 mg/kg body weight and group IV treated with 100 mg/kg body weight of gallic acid, respectively as compared to control group. The group V and VI treated with 100 mg/kg body weight of gallic acid + 25 mg/kg body weight of acetylsalicylic acid, and 100 mg/kg body weight of gallic acid + 50 mg/kg body weight of acetylsalicylic acid exhibited significant elevation in the specific activity of DTD by 2.41 fold (*p* < 0.001) and 2.64 fold (*p* < 0.001) as compared to control group, respectively. Group V showed enhancement in the specific activity by 1.95 fold (*p* < 0.001) as compared to 25 mg/kg body weight of acetylsalicylic acid treated group. Group VI showed enhancement in the specific activity by 1.46 fold (*p* < 0.001) and 1.10 fold (0.05) as compared to 50 mg/kg body weight of acetylsalicylic acid and 100 mg/kg body weight of gallic acid, respectively. The combined inductive effects of gallic acid and acetylsalicylic acid were higher on the specific activities of both of these enzymes than their individual effects. These results are detailed in Table 2.

### Superoxide dismutase and catalase

The significant enhancement in the specific activity of SOD was found in the group IV treated with gallic acid (100 mg/kg body weight) alone by 1.47 fold (*p* < 0.01) and also in group V and VI treated with 100 mg/kg body weight of gallic acid + 25 mg/kg body weight of acetylsalicylic acid, and 100 mg/kg body weight of gallic acid + 50 mg/kg body weight of acetylsalicylic acid by 1.48 fold (*p* < 0.01) and 1.58 fold (*p* < 0.001), respectively as compared to control group. The animals from group V showed enhancement in the specific activity by 1.37 fold (*p* < 0.001) as compared to 25 mg/kg body weight of acetylsalicylic acid. Group VI exhibited enhancement in the specific activity by 1.34 fold (*p* < 0.01) as compared to 50 mg/kg body weight of acetylsalicylic acid. In case of specific activity of catalase, there was significant elevation in the animal from group IV treated with gallic acid alone by 1.42 fold (*p* < 0.001), group V treated with 100 mg/kg body weight of gallic acid + 25 mg/kg body weight of acetylsalicylic acid by 1.47 fold (*p* < 0.001), and group VI treated with 100 mg/kg body weight of gallic acid + 50 mg/kg body weight of acetylsalicylic acid by 1.85 fold (*p* < 0.001) as compared to control group. Group V showed enhancement in the specific activity by 1.41 fold (*p* < 0.001) as compared to 25 mg/kg body weight of acetylsalicylic acid treated group. Group VI showed enhancement in the specific activity by 1.63 fold (*p* < 0.001) and 1.30 fold (0.001) as compared to 50 mg/kg body weight of acetylsalicylic acid and 100 mg/kg body weight of gallic acid, respectively. The combined inductive effects of gallic acid and acetylsalicylic acid were higher than their individual effects on the specific activities of both of these enzymes. The results are shown in Table 3.

**Table 3:** Modulatory effects of gallic acid, acetylsalicylic acid and their combinations on specific activities of the hepatic antioxidant enzymes and magnitude of peroxidative damage in mice

Groups→ Parameters ↓	Control	25 mg/kg acetylsalicylic acid	50 mg/kg acetylsalicylic acid	100 mg/kg gallic acid	100 mg/kg gallic acid + 25 mg/kg acetylsalicylic acid	100 mg/kg gallic acid + 50 mg/kg acetylsalicylic acid
SOD (μmole/mg protein)	4.831 ± 0.26 (100)	5.201 ± 0.29 (107.66)	5.710 ± 0.43 (118.19)	7.115 ± 0.47^#^ (147.77)	7.157 ± 0.22^#£^ (148.14)	7.675 ± 0.30^§ǂ^ (158.85)
CAT (μmole H_2_O_2_ consumed/min/mg protein)	20.175 ± 0.54 (100)	20.975 ± 1.30 (103.96)	22.875 ± 0.63 (113.38)	28.675 ± 1.18^§^ (142.13)	29.705 ± 0.26^§£^ (147.21)	37.325 ± 0.45^§ȣɸ^ (185.00)
GR (nmole of NADPH consumed/min/mg protein)	41.157 ± 1.87 (100)	44.748 ± 0.88 (108.72)	49.517 ± 2.18 (120.31)	59.164 ± 2.18^§^ (143.74)	60.450 ± 2.13^§£^ (146.87)	64.951 ± 2.36^§ȣ^ (157.81)
GPx (nmole of NADPH consumed/min/mg protein)	19.473 ± 1.47 (100)	20.349 ± 2.49 (104.49)	30.459 ± 1.59^§^ (156.41)	31.761 ± 1.16^§^ (163.09)	32.572 ± 1. 20^§£^ (167.26)	33.419 ± 1.71^§^ (171.61)
Peroxidative damage (nmole MDA formed/mg of protein)	1.029 ± 0.037 (100)	0.828 ± 0.048 (80.50)	0.782 ± 0.056^*^ (76.03)	0.680 ± 0.029^§^ (66.13)	0.559 ± 0.052^§€^ (54.31)	0.611 ± 0.0663^§^ (59.42)

Gallic acid and acetylsalicylic acid were given alone and in combinations through oral gavage for two weeks at desired doses (milligrams of per kilogram of body weight) as mentioned in the table. Abbreviations: *CAT* catalase, *SOD* superoxide dismutase, *GR* glutathione reductase, *GPx* glutathione peroxidase, *MDA* malondialdehyde. Values are expressed as mean ± SEM of 5–6 animals. Values in parentheses are presented as percent of control.^*^(*p* < 0.05), ^#^(*p* < 0.01) and ^§^(*p* < 0.001) represent significant changes relative to control. ^€^(*p* < 0.05) and ^£^(*p* < 0.001) represent significant changes relative to 25 mg/kg body weight of acetylsalicylic acid. ^ǂ^(*p* < 0.05) and ^ȣ^(*p* < 0.001) represent significant changes relative to 50 mg/kg body weight of acetylsalicylic acid. ^ɸ^(*p* < 0.001) represents significant changes relative to 100 mg/kg body weight of gallic acid

### Glutathione reductase and glutathione peroxidase

The animal groups IV, V and VI treated with 100 mg/kg body weight of gallic acid, 100 mg/kg body weight of gallic acid + 25 mg/kg body weight of acetylsalicylic acid, and 100 mg/kg body weight of gallic acid + 50 mg/kg body weight of acetylsalicylic acid showed augmentation in the specific activity of GR by 1.43 fold (*p* < 0.001), 1.46 fold (*p* < 0.001) and 1.57 fold (*p* < 0.001), respectively as compared to control group. Group V showed enhancement in the specific activity by 1.13 fold (*p* < 0.001) as compared to 25 mg/kg body weight of acetylsalicylic acid. Group VI exhibited enhancement in the specific activity by 1.31 fold (*p* < 0.001) as compared to 50 mg/kg body weight of acetylsalicylic acid. In case of GPx, the specific activities in the animal group III, IV, V and VI treated with 50 mg/kg body weight of acetylsalicylic acid, 100 mg/kg body weight of gallic acid, 100 mg/kg body weight of gallic acid + 25 mg/kg body weight of acetylsalicylic acid, and 100 mg/kg body weight of gallic acid + 50 mg/kg body weight of acetylsalicylic acid showed increase by 1.56 fold (*p* < 0.001), 1.63 fold (*p* < 0.001), 1.67 fold (*p* < 0.001) and 1.71 fold (*p* < 0.001), respectively as compared to control. Group V showed enhancement in the specific activity by 1.60 fold (*p* < 0.001) as compared to 25 mg/kg body weight of acetylsalicylic acid treated group. The combinatorial effects of gallic acid and acetylsalicylic acid in the induction of specific activities of GR and GPx, were higher than their individual effects. These results are depicted in Table 3.

### Peroxidative damage

In the present study, peroxidative damage was found to be significantly decreased in the animals from group III, IV, V and VI treated with 50 mg/kg body weight of acetylsalicylic acid, 100 mg/kg body weight of gallic acid, 100 mg/kg body weight of gallic acid + 25 mg/kg body weight of acetylsalicylic acid, and 100 mg/kg body weight of gallic acid + 50 mg/kg body weight of acetylsalicylic acid by 24.00% (*p* < 0.05), 33.91% (*p* < 0.001), 45.67% (*p* < 0.001) and 40.62% (*p* < 0.001), respectively as compared to control group. The animal group V exhibited significantly decreased peroxidative damage by 32.48% (*p* < 0.01) as compared to 25 mg/kg body weight of acetylsalicylic acid treated group. These results are detailed in Table 3.

### Bodyweight and diet consumption

Animals from each group have been recorded for their body weight and diet consumption to check any possible adverse effect of gallic acid and acetylsalicylic acid treatment. There were no significant changes observed in body weight and, food and water consumption (data not shown) during the experiment.

## Discussion

*S. aegyptiaca* has been known for its beneficial effects since long, and ascribed the same mainly to salicylate compounds. This medicinal plant also contains other active principles with antioxidant properties and expected to contribute to its preventive and curative action against reported pathological conditions [29, 30]. The chemical compounds which behave as an antioxidant are electron donors. Therefore, the reduction potential which is a determinant of ability to donate electron could be closely linked to the protective action of an antioxidant in biological system. The negative values of redox potential probably enabled these active principles to act as an antioxidant and in turn scavengers of free radicals which reflected from the results of DPPH assay. Importantly, the relative efficacy of antioxidant activity was similar to their relative order of redox potential. It was significant that acetylsalicylic acid has shown lowest redox potential and antioxidant activity. These findings suggest that the phytochemicals such as gallic acid, quercetin, rutin and vanillin, other than salicylates also contribute to the medicinal properties of *S. aegyptiaca,* since acetylsalicylic acid showed lowest lowest antioxidant activity among these phytochemicals.

Inflammation was reported to be closely linked to initiation and progression of various diseases [31–33]. Many anti-inflammatory agents reported to prevent heat induced denaturation of proteins [34] which could be used to test anti-inflammatory activity of the drugs [35]. In the present study, therefore, the exhibition of preventive effect against the heat induced denaturation of proteins by all the five phytochemicals tested was suggestive of the possibility to possess the anti-inflammatory activity (Fig. 2). Interestingly, acetylsalicylic acid which showed the lowest free radical scavenging activity, measured by DPPH assay, was most effective among these phytochemicals to prevent the denaturation of proteins. The heat is physical agent and expected to have different mode of action compared to free radical mediated damage.

To find the relevance of redox potential, the antioxidant activity of phytochemicals was examined in biological system. In *ex vivo,* the oxidative damage and protein carbonyl damage were determined in microsomes and cytosolic protein respectively prepared from liver of mice. The oxidative damage is known to be initiated by hydroxyl free radical (HO˙) and propagated mainly peroxyl free radical (ROO˙). Inactivation of these free radicals is expected to inhibit the peroxidation. In the present study, except vanillin, all the phytochemical tested significantly inhibited the peroxidation in dose dependent manner (Fig. 4). Importantly, this order of their protective action was similar to that of redox potential which suggested the close link between these two properties of phytochemical. This possibility was supported by the similar pattern of protective action exerted by the phytochemicals against the cytosolic protein damage initiated by these OH˙ radicals (Fig. 3).

It may be mentioned that ability of the antioxidant to donate electron could be modulated. In the microchemical environment, the interaction or binding of antioxidant with the surrounding molecules could augment or lower its ability to donate the electron to the acceptors like free radicals resulting into enhanced or lowered protective effect respectively. Sometimes, under certain conditions, the antioxidant might reverse its electron donation property and becomes electron acceptor and in turn oxidant [36]. In case of vanillin, therefore, it was possible that some chemical agent in the present *ex vivo* studies might have influenced its redox potential affecting its antioxidant property and on the other hand enhanced its potential which protected protein from denaturation induced by energy in the form of heat.

Efficacy of these phytochemicals in animal system was studied using gallic acid and acetylsalicylic acid as representatives which showed the higher antioxidant and anti-inflammatory activity respectively. Mice were treated with gallic acid and acetylsalicylic acid alone or in combination for two weeks and their effect on the phase I and phase II enzymes which are known to cause the detoxification of chemicals detrimental to living system through the process of oxidative activation, deactivation and promotion of their elimination from the body [19]. Modulatory effect of gallic acid and acetylsalicylic acid was also studied on the free radical metabolizing enzymes which render the protection against oxidative stress, in addition to the status of the oxidative damage in the liver of mice.

Cytochrome P450 reductase and cytochrome b5 reductase are important members of the phase I enzyme system whose specific activities were found to be enhanced with the treatment of gallic acid and acetylsalicylic acid alone or in combination. Under such conditions the toxic chemicals or drugs would be metabolised and converted these two hydrophyllic metabolites which serve as substrate to phase II enzymes namely glutathione-S-trasferase and DT-Diaphorase. Expectedly, both the phytochemicals gallic acid and acetylsalicylic acid augmented the specific activities of phase II enzyme system, as a result the polarity and hydrophobicity produced by the action of phase enzymes would likely to be increased further and in turn accelerated their removal from the body [37]. DT-Diaphorase also functions as antioxidant enzyme [20]. Apart from facilitating the two electron reduction of many xenobiotics inducing carcinogenesis [38], it reduces quinones to prevent the formation of semi quinones and protect from its reactive intermediate metabolites. These findings of the present work suggested that gallic acid and acetylsalicylic acid have a chemopreventive ability and likely to protect against the detrimental effects of toxic compounds. It may be mentioned that combination of gallic acid and acetylsalicylic acid were relatively more effective as compare to their individual action.

Free radicals generated during the normal metabolic activity or a consequence of detrimental effect of toxic compounds, react with biomolecules and affect their structure resulting into oxidative damage which could be, expressed as metabolic impairment and cell death. As a preventive measure against oxidative stress, organisms have evolved endogenous defence. SOD, one of the enzymes of defence system dismutates O_2_^−^ to H_2_O_2_ which is subsequently removed by catalase and GPx by reducing it to H_2_O [39]. GR is another important antioxidant enzyme that catalyses NADPH dependent reduction of glutathione disulphide (GSSG) to glutathione and maintains its level in the cell for the antioxidant functions [40]. Both the phytochemicals gallic acid and acetylsalicylic acid enhanced the specific activities of SOD, catalase, GPx and GR significantly, as consequence O_2_^−^ likely to be dismuted and H_2_O_2_ thus formed be reduced to H_2_O resulting into protection against oxidative stress.

In a cell, the one electron reduction of H_2_O_2_ catalysed by transition metals generates HO˙, the most reactive oxygen species (with biological half-life 10^− 9^ s) which interacts with biomolecules by abstracting the hydrogen and subsequently breaking the chemical bond hemolytically [41]. As mentioned earlier, HO˙ initiates free radical chain reaction in the form of peroxidation. Any phytochemical which could react with these free radicals involved in initiation (HO˙) and propagation (peroxyl) of peroxidation would inhibit the oxidative stress. The protective action exhibited by gallic acid and acetylsalicylic acid against oxidative damage in the present study confirm their ability to scavenge the free radicals and in turn their antioxidant activity.

## Conclusion

In the present work, the electron donating ability, antioxidant potential and biological activity are found to be closely interlinked. Among the used phytochemicals gallic acid, quercetin, rutin and vanillin appeared to be better antioxidants and might be mainly contributing to antioxidant property of *S. aegyptiaca* rather than salicylates as acetylsalicylic acid showed lowest electron donation ability and antioxidant activity. However, acetylsalicylic acid exhibited highest anti-inflammatory activity, hence playing a role in contributing to its medicinal property. Augmentation of the specific activity of phase I and phase II as well as antioxidant enzymes and inhibition of oxidative damage by gallic acid and acetylsalicylic acid in the liver of C57BL/6 mice suggested that these phytochemicals are likely to behave as effective chemopreventive agents by enhancing the metabolisation and antioxidant status of the animals. These phytochemicals, in particular gallic acid and acetylsalicylic acid being antioxidant and anti-inflammatory agents respectively, may be further explored for their preventive role against oxidative stress and inflammation leading diseases including several cancer.

## Abbreviations

BHT: 3,5-Di-*tert*-4-butylhydroxytoluene
CDNB: 1-chloro-2,4-dinitrobenzene
DCPIP: 2,6-dichlorophenol-indophenol
DPPH: 1,1-Diphenyl-2-picrylhydrazyl
DTD: DT-diaphorase
DTNB: 5,50-dithiobis-2-nitrobenzoic acid
EDTA: Ethylenediamine tetraacetic acid
GPx: Glutathione
GR: Glutathione reductase
GSH: Reduced glutathione
GSSG: Oxidized GSH
GST: Glutathione S-transferase
MDA: Malondialdehyde
NADH: nicotinamide adenine dinucleotide
NADPH: reduced nicotinamide adenine dinucleotide phosphate
ORP: Oxidation reduction potential
SOD: Super oxide dismutase
TBA: Thiobarbituric reduced
TBARS: Thiobarbituric acid reactive substances
